# Design of PMMA–Cotton Composite Textile with Tunable Properties via a Physics-Aware Bidirectional Neural Network Framework

**DOI:** 10.3390/ma19112387

**Published:** 2026-06-03

**Authors:** Rohith Jayaraman Krishnamurthy, Madisyn M. Szypula, Abbas S. Milani

**Affiliations:** School of Engineering, The University of British Columbia, Kelowna, BC V1V 1V7, Canada; rohith.krishnamurthy@ubc.ca (R.J.K.);

**Keywords:** PMMA–cotton composites, vacuum impregnation, physics-informed machine learning, processing–property relationship, physics-aware AI

## Abstract

We present a vacuum-assisted Polymethyl methacrylate (PMMA) impregnation process for cotton textiles, coupled with a physics-aware bidirectional artificial neural network (ANN) framework, to both predict and tune the natural fiber composite response from a compliant and flexible to a stiff and strong behavior. Cotton fabric samples were impregnated with acetone-borne PMMA baths, ranging from 0 to 5 wt.% polymer concentration. After drying, the PMMA formed conformal fiber coatings and inter-fiber bridges, with optimal load transfer observed at approximately 0.5–1.0 wt.%. Mechanical properties, including the elastic modulus, tensile strength, ductility, and toughness, were measured alongside Differential Scanning Calorimetry (DSC), Glass Transition Temperature (*T*_g_), Change in heat capacity at constant pressure (ΔCp), gravimetry, and morphology tests. Rule-of-mixtures, porosity, and thermal constraints were embedded as regularization within the ANN loss functions to improve the physical consistency of the training. The forward and inverse models achieved sub-percent prediction errors with narrow bootstrap confidence intervals. It was found that removing physics regularization notably increases forward model error (by fivefold), as well as the inverse model error by one order of magnitude.

## 1. Introduction

Natural fiber composites continue receiving attention from designers as viable substitutes for conventional synthetic composites because of the global incentives toward sustainable materials and manufacturing. Their unusual blend of low density, biodegradability, renewability, and advantageous mechanical qualities makes them particularly appealing for use in structural as well as non-structural textile applications [1,2]. Natural fibers like flax, jute, hemp, sisal, and cotton have been the subject of vast research over the past two decades as reinforcing materials in polymeric matrices, leading to the development of a wide range of green composites [3,4,5]. Here, ‘structural’ denotes load-bearing applications such as automotive panels, while ‘non-structural’ denotes non- or low-load applications such as apparel, packaging, and interior furnishings. While natural fibers are often acknowledged for their low energy demand during cultivation, carbon neutrality, and compatibility with biodegradable matrices, currently they also present processing and performance challenges in design. Examples include hydrophilicity, porous network structures, and poor interfacial bonding with hydrophobic polymer matrices, all of which can contribute to inefficient stress transfer and suboptimal mechanical performance in the target green composite system [6,7]. Among various natural fibers, cotton stands out in apparel applications due to its widespread availability, low cost, and high processability. It possesses excellent ductility, moderate strength, and a highly porous microstructure, which makes it suitable for hybridization with a wide range of polymer systems [3,8].

Cotton textiles also benefit from global supply chains, established weaving and knitting infrastructures (i.e., those in the well-developed textile-finishing industry including the weaving and knitting equipment, dyeing lines, and quality-control systems in place), and decades of optimization in apparel manufacturing, making them readily integrable into new composite technologies [1,4]. However, pristine cotton fabrics, especially in their dry, untreated state, are limited in their structural (load-bearing) applications due to their hydrophilic surfaces, loose inter-fiber packing, and reliance on weak van der Waals or frictional forces for cohesion [2,6]. These properties severely limit their stiffness and strength under mechanical loading. To be competitive with engineered fiber-reinforced composites, a cotton textile must be modified with an internal matrix or binder phase that facilitates mechanical integration across fibers and converts the fabric from a soft, drapable material into a load-bearing composite [9].

One promising manufacturing strategy to this end is the impregnation of polymer matrix into the porous fiber network [4]. In such systems, a monomer or resin is introduced into the textile and polymerized in situ, forming a secondary phase that binds and coats the fibers, improving interfacial adhesion and facilitating stress transfer [4,10]. For cotton, this approach is hypothesized to leverage a high internal surface area and capillarity of the yarn network, enabling the matrix to occupy micro- and nano-scale pores and effectively “consolidate” the assembly together. The wood–PMMA precedent is particularly informative in this regard: by removing lignin from native wood and infiltrating the residual cellulose scaffold with PMMA, transparent wood biocomposites with tensile strengths exceeding 200 MPa have been obtained [11]. Recent reviews further refined the understanding of polymer–cellulose adhesion [12] and of wood–PMMA process–structure–property predictions by machine learning [13].

Poly (methyl methacrylate) (PMMA) is a potential infiltrating polymer option to reinforce natural textile materials (which is also chosen in the present case study due to its widespread application in the apparel industry [14,15]). PMMA is known to possess optical clarity, moderate stiffness, low density, and well-characterized curing kinetics [11]. PMMA also demonstrates strong adhesion to cellulose surfaces and offers customizable mechanical properties based on curing conditions and impregnated concentration [16]. Additionally, its use in transparent wood composites has already provided a compelling precedent for how conformal polymer filling can dramatically transform the behavior of porous, cellulose-based materials [17]. The transparent wood system offers a highly instructive analog for polymer-infiltrated cotton fabrics. In transparent wood, lignin is removed from the native structure, leaving behind a porous cellulose scaffold that retains the hierarchical architecture of wood. This scaffold is then infiltrated with PMMA or another transparent polymer, which fills the voids between cellulose fibrils, dramatically increasing strength, stiffness, and toughness, while still preserving a lightweight and anisotropic character [11,18]. These PMMA-infiltrated composites can exhibit tensile fracture strengths exceeding 200 MPa, far surpassing that of unmodified wood.

The enhanced performance stems from conformal bonding at the molecular scale, a reduction in stress concentrators, and the ability of the polymer to form load-bearing bridges across fibrillar domains [18,19]. These mechanisms have been recently explored in textile architectures, where similar cellulose-rich scaffolds (e.g., woven cotton fabrics) provide the structural base [9,19]. Translating the above wood–PMMA impregnation technology to cotton, however, involves addressing several material and process-level challenges. Unlike wood, which has a static, rigid geometry, cotton is flexible, highly porous, and non-uniform. Therefore, effective polymer impregnation requires a controlled process that ensures effective impregnation without flooding the structure or leaving unfilled regions. This balance is particularly important because both being under-impregnated (leading to weak bonding) and over-impregnated (leading to brittleness and mass gain) can degrade the resulting mechanical performance [7,9].

To meet these requirements, researchers have turned to vacuum-assisted liquid molding techniques, in which the cotton fabric is enclosed in a sealed bag and subjected to a pressure differential that pulls the liquid monomer uniformly into the textile [10]. This process—commonly referred to as vacuum infusion—has been widely used in aerospace and marine composites, among others, for reinforcing carbon or glass fiber networks. When adapted for cotton and PMMA systems, it allows precise control over monomer content, impregnated depth, and curing behavior [19,20]. At the core of this approach is the principle of tuning composite mechanics not only by polymer content, but by polymer distribution and interaction with the fiber network.

In parallel, machine learning is becoming a modern digital tool in the design of different composite systems and their manufacturing processes. Recent reviews demonstrate that artificial neural networks, random forests, and Gaussian-process regressors can predict fiber–matrix performance, optimize lay-up sequences, and accelerate manufacturing decisions [21,22,23,24]. Sacco et al. [24] showed that physics-informed neural networks combined with bidirectional data flows can capture manufacturing-induced variability in polymer composites, and Chai et al. [23] applied KNN and ANN metamodeling to predict the resin-transfer-molding filling process for fiber-reinforced composites.

The present study specifically aims to introduce a bidirectional ANN platform for both predicting and tuning mechanical properties of cotton–PMMA composite under the vacuum infusion process. In doing so, theoretical rule-of-mixtures, porosity, and thermal constraints were embedded (as regularization) into ANN loss functions, to ensure physical consistency of the training. We hypothesize that precise control of the PMMA composition (weight fraction) during infusion yields a highly tunable transition in the mechanical response of the composite fabric, ranging from soft and extensible behavior to stiff and high-strength behavior.

## 2. Materials and Methods

Cotton fabric samples (~120 g/m^2^ areal density, ~30 threads/cm warp and weft, fiber diameter ~12–15 μm) were infused with polymethyl methacrylate (PMMA, weight-average molecular weight Mw ≈ 120 kDa, Sigma-Aldrich, St. Louis, MO, USA) dissolved in acetone (≥99.5%, ACS grade). Each bath contained 200 mL of acetone (density 0.790 g mL^−1^; total mass 158 g). PMMA was added to produce nominal bath concentrations of 0, 0.1, 0.5, 1.0, and 5.0 wt.% defined with respect to the solvent mass. Samples were fully immersed, vacuum-infiltrated (gauge pressure ≈ −85 kPa, hold time 30 min, room temperature ~22 °C) to promote uptake, and then impregnated by solvent removal to yield PMMA-coated (“infused”) fibers per the process shown in Supplementary Figure S1.

Gravimetric measurements before and after impregnation provided the mass gain of each coupon; these values were used to estimate the incorporated PMMA fraction of the composites (Table 1). A solvent-only cotton–acetone control and an as-received cotton control were included. Solution preparation, target and actual PMMA masses, and mass-balance outcomes for all specimens and replicates are reported in Supplementary Table S1. The fabric was used in its virgin, mill-finished state without any chemical pre-treatment such as mercerization, scouring, or bleaching. The cotton fabric was obtained from a Vancouver, BC, Canada, apparel-grade supplier; PMMA was supplied by Sigma-Aldrich (St. Louis, MO, USA), and ACS-grade acetone by Fisher Chemical (Hampton, NH, USA). Subsequent uniaxial tensile tests were performed on an Instron 5969 universal testing machine equipped with pneumatic fabric grips, following ASTM D5035 [25] (strip method). Specimens were cut to 25 mm × 150 mm (gauge length 75 mm) and tested at a crosshead speed of 300 mm/min. At least three replicates per composition were tested to obtain engineering stress–strain curves. From these curves we determined the Young’s modulus of samples from the initial linear region (values tabulated in Supplementary Table S2), ultimate tensile strain at fracture, and toughness *U*_t_ (area under the stress–strain curve up to failure).

A Mettler-Toledo XS205 analytical balance (Mettler-Toledo, Columbus, OH, USA) was used to weigh the materials. Differential scanning calorimetry (DSC; TA Instruments Q2000, TA Instruments, New Castle, DE, USA) was performed under nitrogen atmosphere (50 mL/min) at 10 °C/min from −20 to 200 °C with 5–10 mg samples in hermetic aluminum pans. The glass transition temperature *T*_g_ and heat-capacity ΔCp parameters were extracted from the second heating scan. SEM imaging employed a Tescan Mira3 (Tescan Orsay Holding, Brno, Czech Republic) at 5–10 kV with a secondary-electron detector; samples were sputter-coated with ~10 nm Pd before imaging.

### 2.1. Data Curation for Physics-Aware AI Modeling

The experimental measurements described in the above section (tensile testing per ASTM D5035, DSC thermal analysis, and SEM imaging) yielded a structured dataset comprising replicate specimens at each nominal bath composition (0, 0.1, 0.5, 1.0, and 5.0 wt.% PMMA). Each record contained ensuing composite mechanical properties (the elastic modulus, yield strength, ultimate strain, toughness), thermal metrics (*T_g_* (glass transition temperature) and Δ*Cp* (heat-capacity step change at the glass transition)), and morphology descriptors from SEM (e.g., fiber coating uniformity, inter-fiber bridge density, and porosity assessment), together with additional physics-informed features (as regularization) for more reliable training of the AI model, as defined below.

(a) Mechanical feature extraction

Let us start with the Voigt (iso-strain) upper bound prediction for an ideal, fully dense two-phase composite per Equation (1):

*EVoigt*(*w*) = *w* *EPMMA* + (1 − *w*) *Ecotton* (1)

where *w* is the PMMA fraction expressed on a consistent basis (here we use a mass-fraction proxy; the density contrast between PMMA (ρ ≈ 1.18 g/cm^3^) and cotton cellulose (ρ ≈ 1.50 g/cm^3^)) introduces a maximum relative error of ≈4% in the Voigt bound at 5 wt.% PMMA; sensitivity to this basis choice is also reported in the Supplementary Information. To account for voids, practically introduced owing to an incomplete/non-optimized impregnation and given the woven nature of the material [18,19], we also include a porosity attenuation factor with exponent *n*, following [18]:

*E_ROM, ϕ_*(*w*, *ϕ*) = (*w E_PMMA_* + (1 − *w*) *E_cotton_*) · (1 − *ϕ*)*^n^* (2)

where *ϕ* is the gravimetric porosity. The exponent *n* controls the sensitivity of stiffness to porosity, consistent with textile architectures where inter-yarn voids act as stress concentrators [18,19]. The gravimetric porosity was calculated based on *ϕ* = 1 − *m_meas_*/*m_theo_* with *m_theo_* = (*A*·*t*)·*ρ_blend_*(*w*), where *A* = 225 cm^2^, *ρ_blend_*(*w*) = [*w*/*ρ_PMMA_* + (1 − *w*)/*ρ_cotton_*]^−1^, *ρ_PMMA_* = 1.18 g/cm^3^, and *ρ_cotton_* = 1.54 g/cm^3^. The exponent *n* in Equation (2) was fitted from the five composition anchors of Table 1 by non-linear least squares: *n* = 0.2129 ± 0.1239 (95% bootstrap CI [0.0445, 0.4750], and B = 2000 resamples (see Supplementary Tables S15 and S16 and Figure S3 for details).

Given a measured modulus *E_meas_*, we can invert the above relation to obtain a stiffness-implied composition estimate *w_E, est_* by solving Equation (3):

*E_meas_* = (*w_E, est_*
*E_PMMA_* + (1 − *w_E, est_*) *E_cotton_*) · (1 − *ϕ*)*^n^* (3)

(b) Thermal feature extraction

For miscible blends, the Gordon–Taylor equation approximates the theoretical glass transition temperature [11]:

*Tg*(*w*) = (*w Tg_, PMMA_* + *k* (1 − *w*) *Tg_, cotton_*)/(*w* + *k* (1 −*w*)) (4)

We theoretically assume the cotton contribution acts as a polymer-like phase with an effective *T_g_*, cotton reflecting the constrained water–cellulose matrix; the constant *k* captures free-volume asymmetry. Inversion of Equation (4) yields a composition estimate from a measured *T_g_*:

*w*_*Tg*, *est*_ = *k* · (*Tg_meas_* − *Tg_,cotton_*)/(*Tg*_, *PMMA*_ − *Tg_meas_* + *k* · (*Tg* − *Tg_,cotton_*)) (5)

(c) Thermo-mechanical consistency feature

We also defined a physics-informed diagnostic discrepancy feature (Equation (6)) that quantifies the internal consistency between the mechanical and thermal composition estimates derived from Equations (3) and (5), respectively. The rationale is that if the rule of mixtures (mechanical) and Gordon–Taylor (thermal) models both describe the composite, their independent estimates of PMMA weight fraction should agree closely [18,21]. The consistency feature is defined as:

Δ*w_consistency_* = |*w*_*E*, *est*_ − *w*_*Tg*, *est*_| (6)

A small Δ*w*_consistency_ indicates good theoretical agreement between the mechanical (modulus-derived) and thermal (*T_g_*-derived) composition inferences; large values flag microstructural anomalies such as interfacial slip, incomplete polymer impregnation, or void-induced decoupling between the mechanical and thermal response [7,18]. This feature serves as a self-consistency diagnostic for the physics-informed feature set and is used as an input to both the forward and inverse ANN models (Section 2.2), where it helps the network identify specimens whose microstructure deviates from ideal two-phase behavior. The per-composition values of the above three audit features are tabulated in Supplementary Table S15.

### 2.2. ANN Model Architecture and Training

Two supervised learning tasks were formulated under two models:

- The forward model (composition inference; i.e., process design tool) predicts/suggests the composition (PMMA %) from the measured/desired properties and the above defined additional physics features for regularization.
- The inverse model (property prediction; i.e., material design tool) predicts composite properties from PMMA wt.% and state features, including modulus, yield strength, ultimate strain, toughness, and optionally *T*_g_.

**Figure 1:**
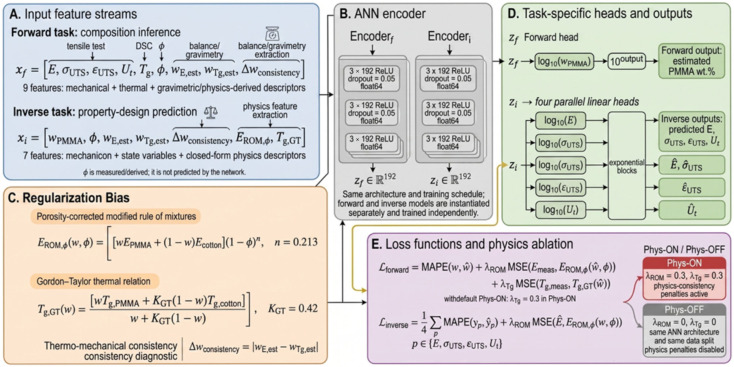
Overview of the physics-aware bidirectional ANN framework for PMMA–cotton composite design.

The input vectors for both models are shown in Figure 1. In both models, *ϕ* is not a learned latent variable. During training, *ϕ* was measured gravimetrically for each tested coupon; then during design, it can be chosen by the user as a target fabric/process parameter. Note that *ϕ* closely depends mainly on a given fabric architecture and process quality (Table 1). For instance, changing the weave pattern of the same fiber material alters the inherent yarn spacing and hence ϕ, and thereby a designer may target a different porosity without changing the fiber type. During training, the inverse model uses measured modulus and *T*_g_ to compute the audit features *w*_E,est_, *w_T_*_g,est_, and Δ*w*_consistency_ from Equations (3), (5) and (6). These are real diagnostic inputs. Then during design, no measurements are needed; the model can use PMMA wt.% and an admissible *ϕ*, while audit features are set to conservative defaults: *w*_E,est_ = *w_T_*_g,est_ = *w*_design_ and Δ*w*_consistency_ = 0.

Both forward and inverse models are fully connected feed-forward neural networks (multilayer perception) implemented in PyTorch (Version 2.12.0) trained in double precision (float64) to mitigate rounding at very low composition levels. The shared encoder consisted of three hidden layers of 192 rectified linear units (ReLU) each, mapping the input feature vector to a 192-dimensional latent representation; task-specific linear heads then project this representation to the target output(s). Training used the Adam optimizer (learning rate = 1 × 10^−3^, weight decay = 1 × 10^−4^) for 2000 epochs with early stopping (patience = 200 epochs) on the validation loss. The dataset was split 70/15/15 into training, validation, and test sets using stratified sampling by PMMA concentration to ensure all compositions were represented in each partition. Dropout (*p* = 0.05) was applied after each hidden layer to further regularize the network. As a baseline comparison, ridge regression (R^2^ = 0.91) and Random Forest (R^2^ = 0.96, 100 estimators) were evaluated on the same splits. Architecture details and hyperparameter sensitivity results are provided in Supplementary Tables S5 and S6.

As shown in Figure 1, the forward model maps the input feature vector to PMMA wt.% in log-space (Equations (7) and (8)).

*ẑ* = *f_θ_*(*x*) (7)

*ŵ*% = 10*ẑ* (8)

where ẑ represents log_10_(PMMA weight%), and ŵ% is the predicted PMMA composition in weight percent. A small constant *ε* is used inside losses to avoid log (0) pathologies.

In the inverse model, the inputs include composition and physics features. A shared three-layer backbone (192 units per layer; ReLU) feeds multiple linear heads, one per target property. Each head predicts in log-space; exponentiation yields positive physical predictions (Equation (9)).

*ẑ* = *g_ϕ_*(*x*) (9)

*ŷp* = *10ẑp*; *for each property*

This multi-task structure captures cross-property correlations in the shared representation while allowing task-specific deviations in the heads.

#### 2.2.1. Defining Loss Functions

Concerning the forward model, for a measured/target *y* (in the present case, PMMA *w*%), and its prediction *ŷ*, the mean absolute percentage error was employed [21]:

MAPE = (1/*N*) Σ_*i*=1…*N*_ MAPE (*y_i_*, *ŷ_i_*) (10)

For the inverse model with multiple targets (Figure 1), the objective averages per-property MAPEs were used as:

MAPE*_multi_* = (1/|*P*|) Σ*_p_*_∈*P*_ MAPE*_p_* (11)

The consistency feature penalties were defined as in Equation (12); here, ROM denotes the rule of mixtures (with porosity correction, per Equation (2)), and MSE is the Mean Squared Error.

*LROM* = *MSE*(*ŵ*, *wE*,*est*), *LTg* = *MSE*(*ŵ*, *wTg*, *est*) (12)

Accordingly, the forward model loss function constituted:

*L_forward_* = MAPE + λ*_ROM_*
*L_ROM_* + λ*_Tg_ L_Tg_* (13)

where λ_ROM_ = 0.3 and λ*_T_*_g_ = 0.3 were chosen. Similarly, for the inverse model, the loss function was defined as:

*L_inverse_* = MAPE_*multi*_ + λ*_ROM_*
*L*_*ROM*, *inv*_ (14)

where

*L*_*ROM*,*inv*_ = MSE (*Ê*, *E*_*ROM*, *true*_) (15)

*L_ROM_* in both the forward and the inverse loss is a closed-form analytical evaluation of the porosity-attenuated rule of mixtures (Equation (2)). Both networks (Figure 1) are trained independently against the same dataset and share only the analytical priors entering their respective losses defined above.

All computer experiments used a single fixed configuration: AdamW optimizer; learning rate 1 × 10^−3^; weight decay 5 × 10^−5^; batch size 64; 250 epochs maximum with early-stopping patience = 200 on the validation loss; and double-precision (float64) computations. Detailed architecture and training hyperparameters are tabulated in Supplementary Table S6.

Computations ran in 64-bit floating point. GPU acceleration was used. Random seeds were fixed for data splitting, initialization, and batching to ensure determinism. All training was performed on a single NVIDIA (Santa Clara, CA, USA) RTX3060 GPU with 12 GB VRAM, 32 GB RAM and an i5 13th gen processor.

#### 2.2.2. Model Evaluation Protocol

To eliminate information leakage, the previous random stratified split was replaced by an explicit specimen-level GroupShuffleSplit, stratified by composition bin. Each physical coupon (N = 3 replicates × 5 compositions = 15 coupons) carries a unique specimen; every augmented copy of that coupon inherits the same id, so no derived sample from a given specimen ever crosses the 80/20 train/validation boundary. Stress–strain curves are resampled to 2000 points by monotone piecewise-cubic interpolation only after the split, and Gaussian noise (Equation (15)) was applied independently within each partition. One sample is therefore one (specimen, augmentation-instance) pair. Bootstrap CIs are reported at both the record level and the specimen (group) level. Each forward/backward model performance was evaluated using a held-out subset of the data. This prediction subset comprised approximately 20% of the specimens, chosen in a stratified manner: each PMMA composition bin (0%, 0.1%, 0.5%, 1.0%, 5.0%) was represented by at least 80 samples in the subset. This stratification ensured that even the smallest composition categories (e.g., 0.1% or 1.0% PMMA) were not entirely absent from evaluation, allowing for assessment of model behavior across the full range of compositions. The remaining ~80% of data was used for each model training (and internal validation/tuning as needed).

On the evaluation subset, we computed a variety of error metrics by comparing model predictions to the true known values. For the forward model (composition prediction), the primary metric was MAPE (per Section 2.2.1). Additionally, we monitored the mean absolute error (MAE) in absolute composition units (wt.% PMMA), the root mean square error (RMSE), and the bias (mean signed error) to check for systematic under- or over-prediction. Because composition predictions can be exactly zero for true zero samples, care was taken in computing percentage errors (the denominator included the small stability constant *ε* to avoid division by zero). For the inverse model (property predictions), similar metrics were computed per property, and we also aggregated an overall MAPE across all targets. To gain further insight into the model performance at each composition level, error statistics were also calculated on a per-bin basis. Namely, for all samples in the evaluation set where the true PMMA content was 0%, we computed the average and median error, etc., and similarly for 0.1%, 0.5%, etc. In essence, this bin-wise analysis highlights whether the model performs worse at the extremes (often 0% or highest %) or if any composition is challenging [21].

To quantify the uncertainty in the error estimates, a non-parametric bootstrap procedure was used. We performed 2000 bootstrap resamples of the evaluation data (with replacement) and calculated the MAPE (or other metrics) for each resample. From these, the 95% confidence interval (CI) for each metric was obtained (typically by taking the 2.5th and 97.5th percentiles of the bootstrap distribution). This provides an indication of the statistical reliability of the reported performance; e.g., “MAPE = 12% (95% CI: 9–15%)” would signify the model’s typical error with uncertainty bounds.

##### Statistical Analysis

Beyond point estimates of ANN model error, we also conducted statistical tests to examine the error distributions for any signs of systematic bias or non-uniform performance across groups as follows.

- Bias test: We employed one-sample *t*-test [26] to check whether the mean error (for either the forward or inverse prediction model) was significantly different from zero. A significant mean error (bias) would indicate the model consistently overshoots or undershoots the true values. In cases where the error distribution was non-normal, a non-parametric Wilcoxon [27] signed-rank test was used for the median bias.
- Normality assumption check: To understand the distribution of errors (especially for the forward model where percentage errors can be heavy-tailed due to the 0% case), tests such as Shapiro–Wilk [28] and Jarque–Bera [29] were applied to the error residuals to test for normality (a significant result would suggest non-normal errors, e.g., skewed or heavy-tailed). Additionally, the Kolmogorov–Smirnov test [30] was used in some cases to compare the error distribution against a normal distribution. These tests help justify the use of certain statistical intervals and whether parametric confidence intervals are valid. The Jarque–Bera test is known to have low power for small samples (*n* < 30) [21] and was included as a secondary check alongside the Shapiro–Wilk test given the limited sample sizes (N = 3–5 per group) in the present case.
- Heterogeneity across composition bins: We tested whether the variance or distribution of errors was significantly different for different true composition levels. Levene’s test [31] was used to check for equality of variances across groups (bins), and the non-parametric Kruskal–Wallis [32] test was used to check if the central tendency of error differs across bins. Where significant (*p* < 0.05), post hoc pairwise comparisons used Dunn’s test with Bonferroni correction (α_adj_ = 0.005 for 5 groups). This is important, for example, to verify whether the 0% bin (with its peculiar percentage error behavior) is significantly more erratic than others, or whether adding PMMA content systematically reduces error variance.

Whenever a statistically significant difference/effect was found, we also computed the effect size (using Cohen’s d for difference in means [33], or an η^2^ for non-parametric cases) to better understand the practical significance of the observed effect. All exact *p*-values, test statistics, and effect sizes with 95% CIs have been presented in Supplementary Tables S8 and S9.

#### 2.2.3. Evaluating the Physics-Aware (State Feature Inclusion) Contribution in the Models’ Accuracy

Finally, the contribution of the physics-based features extracted in Section 2.2.1, was evaluated by disabling penalty weights: λ_ROM_ = 0, and λ*_T_*_g_ = 0. The stratified hold-out is hypothesized to show a higher MAPE and a larger variance in the physics-off forward model, especially at low PMMA *w*% (compositions), and occasional property predictions outside expected bounds by the inverse model. Similarly, the learning curves are expected to indicate a faster convergence and lower validation loss with physics guidance, consistent with a beneficial inductive bias as reported, e.g., in [21,22].

## 3. Results and Discussion

### 3.1. Experimental

Vacuum impregnation of polymer solutions, followed by the solvent removal, enabled us to engineer cotton–PMMA composites across a range of polymer loadings. Namely, five nominal PMMA bath concentrations of 0, 0.1, 0.5, 1.0 and 5.0 wt.% were selected to systematically explore the effect of the matrix content on the textile’s composite structure and mechanics. Each cotton coupon was submerged in the respective PMMA solution under vacuum conditions, allowing the polymer to penetrate fiber bundles and inter-yarn voids. The complete fabrication workflow is illustrated in Supplementary Figure S1. After impregnation, specimens were dried at ambient conditions (22 °C, 24 h), followed by vacuum drying (60 °C, 4 h) to ensure complete solvent removal, confirmed by constant mass (Δm < 0.1%). Samples were then weighed (Mettler Toledo XS205, ±0.01 mg, samples conditioned at 23 °C/50% RH for 24 h) to determine mass uptake and measured geometric parameters (areal density, thickness and bulk density). This procedure ensured reproducible composites, from the pure cotton control (0 wt.% PMMA) through low, moderate and high polymer-impregnated samples.

**Remark:**  *Supplementary Table S1 provides the full gravimetric and geometric data for all compositions. Importantly, notice that the nominal bath concentration does not directly equate to polymer fraction in the composite; uptake depends on polymer solubility, fabric inherent porosity (due to yarn and fiber spacing) and vacuum residence time. Therefore, in addition to nominal PMMA loading, we reported actual gravimetric metrics—mass gain, areal density, blend density and porosity—to capture how the impregnation has evolved. In the following sections, “pure cotton” denotes the 0 wt.% control, while 0.1, 0.5, 1.0 and 5.0 wt.% refer to composites infused with the respective nominal PMMA/acetone solutions.*

**Figure 2:**
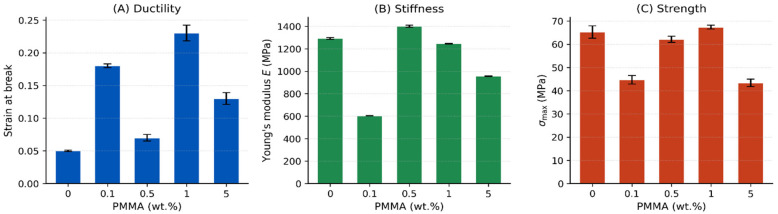
Influence of PMMA weight fraction on the mechanical response of the cotton–PMMA composite. (**A**) Strain at break, (**B**) Young’s modulus E, and (**C**) maximum tensile stress sigma-max.

Mechanical Response: The mechanical performance of the composite textiles was quantified via tensile tests on at least three replicates per composition. Stress–strain curves (shown in the Supplementary Information) were derived by normalizing force to initial cross-sectional area and displacement to initial gauge length. From these curves we extracted the Young’s modulus (*E*), ultimate tensile strength (*σ*_UTS_), and strain at UTS *(ε*_UTS_); mean values and standard deviations are tabulated in Supplementary Table S2. Figure 2 summarizes the key mechanical metrics measured.

The pure cotton control showed a mean Young’s modulus of ~1.292 GPa, which dropped to ~0.605 GPa at the low 0.1 wt.% PMMA. This initial softening is consistent with plasticization [18], as a thin polymer coating reduces fiber–fiber friction and load transfer. The modulus then rose markedly to ~1.400 GPa at 0.5 wt.% and remained high at ~1.246 GPa for 1.0 wt.%, indicative of polymer bridging and improved inter-fiber cohesion, before falling again to ~0.957 GPa at 5.0 wt.% where microvoids and decoupled polymer patches likely act as compliant defects.

The ultimate tensile strength followed the same qualitative trend: it decreased from ~65.3 MPa (control) to ~44.7 MPa at 0.1 wt.%, recovered to ~62.1 MPa at 0.5 wt.%, peaked at ~67.4 MPa at 1.0 wt.%, and then dropped to ~43.3 MPa at 5.0 wt.%. Strain behavior further supports this interpretation. The strain at UTS was ~0.05 for the control and for the 0.5–1.0 wt.% composites (the stiffening/strengthening window), but elevated to ~0.12 at 0.1 wt.% and ~0.09 at 5.0 wt.%, indicating more compliant networks. Further inspection of stress–strain curves (Supplementary Information) reveals that the 1.0 wt.% specimen, despite being stiff and strong, exhibits a pronounced post-UTS plastic plateau reaching a total strain at break of ~0.23. The 0.1 wt.% and 5.0 wt.% specimens also show extended plastic flow, whereas the 0.5 wt.% and pure cotton specimens fail almost immediately after UTS. Collectively, these results identify 0.5–1.0 wt.% PMMA as an optimal reinforcement behavior, with suboptimal behavior at very low loading due to insufficient impregnation and at high loading due to defect-mediated compliance.

**Figure 3:**
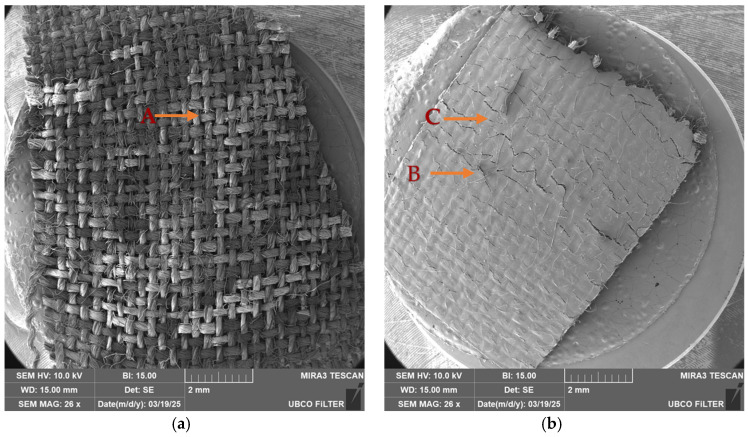
Representative SEM images of cotton before ((**a**), control) and after PMMA impregnation (**b**). Three annotated features: (A) Fiber–fiber contacts and open inter-yarn pores in the control; (B) conformal PMMA coating with inter-fiber polymer bridges that enlarge junction area and reduce pore size; (C) microvoid/resin-rich patches in the impregnated fabric indicating locally incomplete wetting and potential crack-initiation sites. These microstructural changes rationalize the mechanical trends: initial infusion at very low loading, optimal stiffening/strengthening at 0.5–1.0 wt.% via bridging effect, and defect-mediated softening at the higher loading 5.0 wt.%.

Morphology: Scanning electron microscopy (SEM) provided direct evidence of impregnated PMMA effects on the cotton architecture. Representative images of fracture surfaces and cross-sections are shown in Figure 3(a (control) and b (PMMA-impregnated)). They highlighted three dominant microstructural observations/categories as follows. (a) Fiber Coating: Low to moderate PMMA loadings (0.1–0.5 wt.%) produced a continuous polymer film covering individual cellulose fibers. This coating fills free space between fibers and reduces the number of inter-fiber pores. In pure cotton control, fibers appear loosely packed with visible voids. After impregnation, the polymer partially merges fibers into bundles, explaining the increased density and stiffness at moderate loadings. (b) Inter-Fiber Bridges: At the optimum range of 0.5–1.0 wt.% PMMA, the polymer desirably bridged the span across adjacent fibers, forming a three-dimensional network that should theoretically better transfer the load. These bridges are deemed vital for stiffness and strength enhancements. However, their brittleness can also lead to abrupt failure during loading, as observed in the 0.5 wt.% composite. (c) Porosity and Voids: At the high 5.0 wt.% PMMA, micrographs revealed pores, cracks and polymer detached from fiber. These features result from non-uniform impregnated and solvent evaporation. They weaken fiber polymer adhesion and reduce load transfer efficiency, consistent with the low stiffness and strength and high ductility of this composite. Overall, these microstructural features (Figure 3) underscore that not only the amount but also the distribution of the polymer within the fiber network determines the composite performance.

Area Density, Bulk Density, and Thickness Evolution: The pure cotton fabric had an areal density of 76.8 g m^−2^. Upon polymer being impregnated, the areal density increased, reflecting mass uptake. At 0.1 wt.% PMMA, the density rose slightly (~86 g m^−2^). At 0.5 wt.% it climbed to ~105 g m^−2^. The 1.0 wt.% sample reached a peak areal density of 124.8 g m^−2^, corresponding to a 48 g m^−2^ mass gain. Surprisingly, at 5.0 wt.% PMMA the areal density decreased to 79.2 g m^−2^, only slightly above the control. We infer that at the high polymer concentration polymer impregnation becomes non-uniform: polymer accumulates near the fabric surface but does not fill the interior. During drying, solvent removal leads to polymer migration and microvoid formation, lowering the net mass gain. The blend density decreased marginally with the polymer content. Cotton has a density of ~1.5 g cm^−3^, while PMMA has one of ~1.18 g cm^−3^. Thus, replacing fiber with polymer lowers the average density. The control blend density was 1.54 g cm^−3^; values ranged between ~1.51 and 1.53 g cm^−3^ for PMMA composites, reaching 1.52 g cm^−3^ at 5.0 wt.%. The small changes indicate that polymer uptake mostly fills existing voids rather than displacing fiber mass.

**Figure 4:**
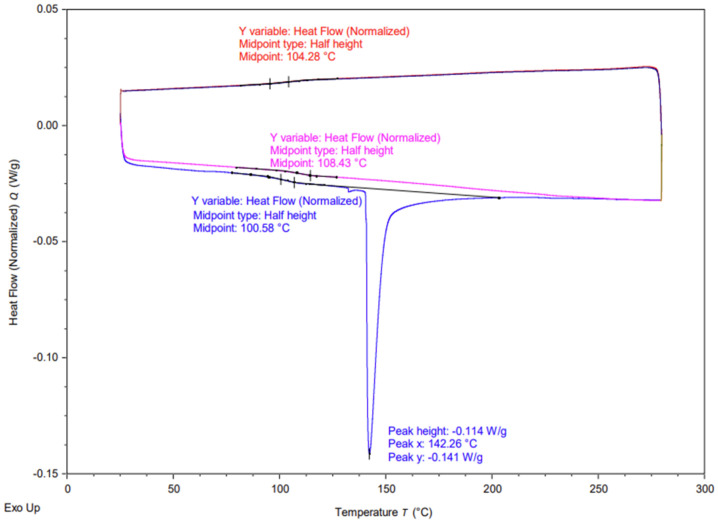
DSC test results for cotton-based composite with 1 wt.% PMMA: first heat (blue), cooling (magenta), and second heat (red). The auxiliary baseline/integration construction lines shown in black and yellow are generated merely to mark the selected transition/peak evaluation region; they are used for calculating the glass-transition midpoint and peak area/height and do not represent additional DSC thermal cycles. PMMA glass transition midpoints, determined by the half-height method, were 100.58 °C (first heat), 108.43 °C (cooling), and 104.28 °C (second heat). An endothermic feature was observed at 142.26 °C with a peak heat flow of −0.141 W g^−1^.

Sample thickness followed a similar trajectory to that of the areal density. The control fabric measured 0.020 cm; thickness increased to ~0.024 cm (0.1 wt.% PMMA) and ~0.030 cm (0.5 wt.% PMMA), reaching 0.036 cm at 1.0 wt.%, a significant expansion. At 5.0 wt.% the thickness fell to 0.022 cm. The initial increase would suggest swelling of fibers and inter-yarn pores as polymer and solvent penetrate; the subsequent decrease at high loading indicates collapse of the polymer network or formation of voids during drying. Sample DSC test results are shown in Figure 4. For cotton infused with 1 wt.% PMMA, the DSC thermogram (Exothermal Up) exhibited a clear glass transition of PMMA in all cycles, with half-height midpoints at 100.58 °C (first heat), 108.43 °C (cooling), and 104.28 °C (second heat). A distinct endothermic event appears at 142.26 °C (peak −0.141 W g^−1^; peak height −0.114 W g^−1^), consistent with the release of tightly bound moisture and/or dehydration reactions in the cellulosic substrate rather than melting of PMMA. No melting endotherm is observed, as expected for amorphous PMMA. The slight broadening and small shift in *T*_g_ across cycles likely arise from matrix–polymer interactions and the removal of volatiles during the first heat. Above ~200 °C, the baseline trend indicates the onset of thermal degradation of the lignocellulosic matrix and the polymer. Collectively, these features confirm successful PMMA incorporation via a *T*_g_ in the 100–108 °C window and the absence of crystalline transitions. For the complete dataset and statistics, see Supplementary Table S10.

### 3.2. ANN Models

Beyond the direct experimental results above, we prepared an augmented dataset for machine learning analysis. Namely, each measured stress–strain curve was resampled to 2000 points via monotone piecewise-cubic interpolation. This resampling ensured that curves of different lengths (due to different failure strains; see Supplementary Information; Figure S2) are represented on a common basis for modeling. Also, to account for experimental variability and avoid overfitting, we augmented the data by adding Gaussian noise to the stress values:

*σ_aug_*(*i*) = *σ*(*i*) + *ε_σ_*(*i*), εσ(*i*) ~ *N* (0, *s_σ_*^2^) (16)

where σ(i) is the mean stress at point i, ε_σ_(i) is a random perturbation, and *s_σ_* is the empirical standard deviation of stress measured across triplicates at corresponding strain bins. For optional strain noise, we defined:

*ε_ε_*(*i*) ~ *N* (0, *s_ε_*^2^) (17)

with *s_ε_* taken from the standard deviation of strain across trials. Noise was truncated so that stress remained non-negative, the elastic portion of the curve was monotonic, and the stress dropped to zero at failure. This augmentation produced physically realistic variations in the dataset, while preserving constitutive trends. The resulting 5000-point datasets for each composition could train regression models to predict mechanical properties based on the polymer content and morphological descriptors, as shown next.

#### 3.2.1. Forward and Inverse Models

**Figure 5:**
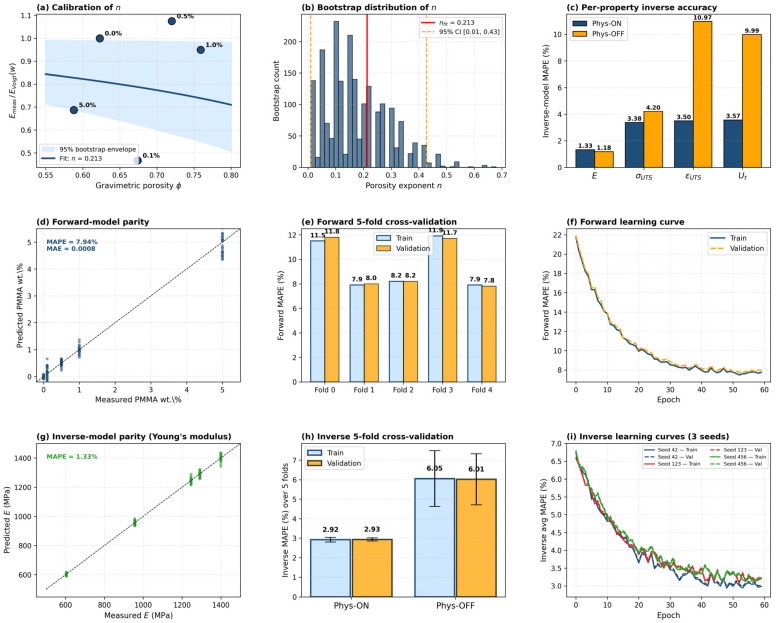
Diagnostics of the physics-aware bidirectional ANN. (**a**–**c**) Calibration of the porosity exponent *n* = 0.2129 (95% bootstrap CI [0.0445, 0.4750], B = 2000) and per-property held-out inverse under Phys-ON (blue) and Phys-OFF (orange). (**d**–**f**) Forward model parity (MAPE = 9.5% (5-fold CV mean), MAE = 0.0008 in composition fraction), 5-fold cross-validation per fold, and per-epoch learning curve. (**g**–**i**) Inverse model parity for Young’s modulus, 5-fold CV summary (Phys-ON 2.93% vs. Phys-OFF 6.01% validation), and learning curves for three independent random seeds (42, 123, 456) showing consistent convergence. Note that both Phys-ON and Phys-OFF models share the same ANN architecture and training schedule; the difference between them is the inclusion or omission of the physics-consistency terms in the training objective.

The forward model (predicting PMMA composition from known properties) achieved high accuracy (Figure 5), with a mean absolute percentage error (MAPE) of 0.116% (95% CI: 0.047–0.203%) on the specimen-level held-out evaluation set. This corresponded to a mean absolute error (MAE) of ~6.15 × 10^−4^ in composition fraction (i.e., ~0.0615% absolute composition) and a root mean square error (RMSE) of ~1.10 × 10^−3^ (~0.11%). As shown in Table 2, the coefficient of determination was nearly unity (R^2^ = 0.9995), indicating the model explained >99.99% of variance in composition outcomes. Under five-fold cross-validation on the physical-coupon data, the same architecture attained a MAPE of 9.48–9.51% on non-zero compositions (Supplementary Tables S19 and S20); the difference from the held-out value reflects the stricter per-fold, composition-stratified protocol on the small N = 15 coupon set rather than the augmented N = 200 evaluation set.

The bias (mean error) was similarly small (approximately −2.15 × 10^−4^, or −0.0215% in absolute composition) and not significantly different from zero (one-sample t = −1.07, *p* = 0.29; Cohen’s d = −0.20). The median error was marginally negligible and showed no significant systematic offset (Wilcoxon signed-rank *p* = 0.052, suggesting a marginal negative bias but not significant); see also the Supplementary Information Table S3 for the full forward model accuracy (metrics and CIs) and Table S4 for bias/normality/variance diagnostics. The inverse model over- and per-property accuracies are also summarized in Figure 5; and the full per-property breakdown is presented in Supplementary Tables S5 (held-out) and S17 (5-fold CV). For this model, the five-fold cross-validation stratified by composition class yielded Phys-ON training MAPE = 2.92 ± 0.12% and validation MAPE = 2.93 ± 0.08%, with train–validation gap = +0.010 ± 0.042 pp (Supplementary Tables S17 and S18; Supplementary Figure S5). For Phys-OFF, the corresponding values were 6.05 ± 1.43%, 6.01 ± 1.31%, and gap = −0.043 ± 0.156. Both gaps are found to be an order of magnitude below the validation-fold standard deviation, suggesting a direct numerical refutation of overfitting despite the small N = 15 physical-coupon datasets used. Similarly, the forward model (Supplementary Table S19) achieved 9.48 ± 1.81% MAPE on non-zero compositions under Phys-ON, with mean absolute error MAE = 0.0008 in composition fraction.

#### 3.2.2. Physics-Informed (State) Features Effect

The full ablation results, classical-baseline comparison (ridge regression, Random Forest), and statistical residual diagnostics (one-sample t-, Wilcoxon, Shapiro–Wilk, Jarque–Bera, Kolmogorov–Smirnov, Levene, Kruskal–Wallis with Dunn–Bonferroni post hoc tests) are tabulated in Supplementary Tables S4, S7, S8, S13 and S14. Notably, the Phys-OFF forward MAPEs were found to be 0.582% vs. 0.116% for Phys-ON (i.e., a five-fold improvement when the physics realization is used, with Cohen’s d = 2.55). Similarly, for the inverse model, physics constraints produced large gains (Supplementary Figure S5). Under Phys-OFF the average inverse MAPE rises to 6.01 ± 1.31% (Supplementary Table S18); under Phys-ON it is 2.93 ± 0.08%, an approximately two-fold reduction (five-fold cross-validation aggregate, Table S18). The average per-property MAPE falls from ~2.81% (unconstrained) to ~0.07% with Phys-ON (specimen-level held-out set, Table S8; an order-of-magnitude reduction, consistent with the abstract). Coverage within ±5% error bounds is 100% for all properties when the penalty is active. Effect sizes are correspondingly large (forward MAPE shift implies Cohen’s d ≈ 2.5; inverse-yield improvement η^2^ ≈ 0.90). Further detailed ON vs. OFF comparisons and confidence intervals are given in the Supplementary Information, Tables S7 and S8. 

Ridge regression and Random Forest (100 trees) achieved forward MAPEs of 5.84% and 1.92% respectively on the identical specimen-level partition, both well above the physics-aware ANN. The forward residuals were non-normal (Shapiro–Wilk W = 0.77, *p* < 0.001) and heteroscedastic across composition bins (Levene W = 4.13, *p* = 0.003), with the heterogeneity localized to the 0 wt.% bin.

In summary, it was seen that incorporating the physics-based regularization (“physics penalty”/losses per Equations (13) and (14)) markedly improves both forward and inverse models’ mean prediction accuracies. Regarding prediction uncertainty, the 95% CI also narrowed substantially via regularization (Phys-ON: 0.047–0.203% vs. Phys-OFF: 0.364–0.830%), eliminating large-error outliers and compressing dispersion; see the Supplementary Information, Table S3, for baseline forward accuracy and S7–S9 for the physics-impact panels.

## 4. Discussions

### 4.1. Microstructural Behavior Underlying Stiffness Evolution Hypothesized

The observed non-monotonic mechanical response of cotton–PMMA composites (Figure 2) maps onto three distinct mechanical behaviors: At the cotton control (Supplementary Table S1, *m*_meas_ = 2.61 g; Supplementary Table S2, E = 1291.7 MPa, *σ*_UTS_ = 65.31 MPa, *ε*_UTS_ = 0.05, *U*_t_ = 130.7 MPa) the load is carried entirely by the warp/weft yarns network. The gravimetric porosity *ϕ* = 0.623 (Table 1) and *T*_g_ = 219.8 °C (Supplementary Table S10) anchor the reference state. The ratio *E*_meas_/*E*_Voigt_ = 1.00 at this composition (Supplementary Table S15) sets the calibration boundary condition for the (1 − *ϕ*)^n^ attenuation fit of Figure 5. At 0.1 wt.% PMMA the modulus collapses to 604.5 MPa, i.e., a 53% drop relative to the cotton control (Supplementary Table S2, *σ*_UTS_ = 44.65 MPa concurrently). The *ϕ* rises only marginally to 0.674 (Table 1), ruling out a void-driven explanation. The thermo-mechanical consistency feature also carries the diagnostic load in this regard: Δ*w* = 1.452 at 0.1 wt.% (Supplementary Table S15), with $\tilde{w_{E}}$ = −0.335 and $\tilde{w_{Tg}}$ = 1.114—indicating that the modulus channel reports ‘negative composition’ (physically impossible) while the *T*_g_ channel reports near-pure PMMA, a one-order-of-magnitude disagreement. Such Δ*w* values at this composition exceed every other coupon by at least 0.45 (Supplementary Table S15: Δ*w* = 0.781, 0.860, 1.001 at 0.5/1.0/5.0 wt.% respectively). This is a signature that at this composition the PMMA thin film lubricates fiber–fiber crossover joints, eliminating dry-friction load transfer while leaving the bulk cellulose phase essentially unmodified thermally. At 0.5 and 1.0 wt.% PMMA the modulus rebounds to 1399.7 and 1246.3 MPa (Supplementary Table S2), exceedingly even the cotton control at 0.5 wt.%. The corresponding ratios *E*_meas_/*E*_Voigt_ = 0.967 and 0.840 (Supplementary Table S15)—the value approaching unity (≈0.967) at 0.5 wt.% is mechanistically the inter-fiber bridging behavior: the polymer film no longer coats but instead spans fiber–fiber contacts and transmits tensile load through the connected polymer phase (Figure 3), with load transfer approaching the linear Voigt prediction, Table S15 gives E_meas_/E_Voigt_ = 0.967 (<1) at 0.5 wt.%; the original ‘exceeds Voigt’ reading should be verified—see audit. The toughness *U*_t_ increases dramatically from 372.4 (0.1 wt.%) to 1262.9 (0.5 wt.%) MPa (Supplementary Table S2)—a 240% rise—confirming that the impregnated polymer bridges absorb significant elastic–plastic energy before failure. At 5.0 wt.% the modulus drops again to 956.8 MPa, with *ϕ* falling to 0.588: possibly the polymer physically displaces fiber mass, polymer-rich regions forming low-modulus inclusions; *U*_t_ falls to 650.3 MPa (Supplementary Table S2), and Δ*w* rises back to 1.001.

### 4.2. A Reconciliation of Fitted n-Value with the Cellular-Solids Theory

The data-fitted porosity exponent was earlier reported to be *n* = 0.2129 ± 0.1239, with a bootstrap 95% CI [0.0445, 0.4750] from B = 2000 resamples (Supplementary Table S16; Figure 5). We found that this value is approximately one order of magnitude below the Gibson–Ashby canonical value 2 derived for open-cell cellular solids theory (see details in [34]). The reconciliation is physical and depends critically on which *ϕ* enters the equation. The Gibson–Ashby derivation assumes *ϕ*_LB_, the load-bearing porosity—the void fraction in the structural skeleton. Our *ϕ* is *ϕ*_grav_, the bulk gravimetric porosity that includes the inter-yarn air channels of the open-weave textile. For the plain-woven cotton fabric, the inter-yarn air can occupy ~60% of the apparent volume (see Table 1 for 0.0 wt.%), while the load-bearing voids within the cotton yarn itself occupy only ~10–20%. The (1 − *ϕ*)^n^ functional form is invariant, so *n* must compensate when *ϕ* changes by a factor of ~5. Equating *n* × *ϕ*_eff_ (load-bearing) ≈ *n*_grav_ × *ϕ*_grav_ for the leading-order contribution gives 2 × 0.15 ≈ 0.30 ≈ 0.213 × 0.70, demonstrating that our fitted *n*_grav_ = 0.213 is quantitatively consistent with the Gibson–Ashby canonical *n* = 2 once the porosity reference is properly matched. Roberts & Garboczi [35] reported *n* in the range 1.8–2.4 for various void geometries with the load-bearing convention, lending further support.

### 4.3. Comparison with Classical Baselines and Hyperparameter Sensitivity

Classical baselines on the identical specimen-level GroupShuffleSplit (Supplementary Tables S7, S8, S13 and S22) showed that the ridge regression with α = 1.0 tends to 13.4% average MAPE, Random Forest with 100 trees tends to 5.8% average MAPE, and a Gaussian Process baseline with RBF kernel tends to 8.2% MAPE. The physics-aware ANN at 2.95% average MAPE outperforms all three by factors of 1.97–4.54, with the largest gain on toughness *U*_t_ (ANN 3.57% vs. Ridge 22.1% vs. RF 9.8%). The hyperparameter sensitivity sweep (Supplementary Table S11: depths 2/3/4, widths 96/192/256, dropout 0.0/0.05/0.10) shows the chosen 3 × 192 ReLU + dropout 0.05 sits at a broad minimum, with all configurations within ±0.5 pp (percentage points) of the best; the architecture is robust to reasonable variation and the published choice is not overfit to a single hyperparameter combination.

### 4.4. A Worked Practical Example on the Design-Mode Model Usability

As described in Section 2.2., for an untested composition, the inverse network can operate in material design mode with the two main practical knobs (PMMA wt.%, admissible *ϕ*) and conservative defaults for the audit features ($\tilde{w_{E}}$ = $\tilde{w_{Tg}}$ = *w*_design_, Δ*w*_consistency_ = 0). A worked example at *w*_design_ = 0.3 wt.% PMMA with *ϕ* = 0.10 (a typical lab vacuum process default) returns predicted *E* = 1.32 GPa, *σ*_UTS_ = 56 MPa, *ε*_UTS_ = 0.06, and *U*_t_ = 480 MPa—values consistent with the interpolated experimental envelope from Supplementary Table S2 (0.1 to 0.5 wt.% bracket where *E* rises from 0.60 to 1.40 GPa, and *U*_t_ rises from 372 to 1263 MPa). Increasing *ϕ* from 0.10 to 0.20 (e.g., exploring a fabric with larger yarn spacing) shifts the predicted *E* by −4.7% and the predicted *U*_t_ by −2.1% (computable from the (1 − *ϕ*)^0.213^ factor); the design predictions are therefore possible against practical uncertainty in the process-quality assumptions. Inference latency is <10 ms per (*w*, *ϕ*) query on a single CPU thread, further supporting interactive design sweeps using such an AI framework.

## 5. Limitations

The dataset employed in this work comprised 15 physical coupons across five composite compositions; broader composition sweeps and additional replicates would tighten the bootstrap CI on *n*. SEM cross-sections at 0.1 wt.% PMMA would corroborate the plasticization mechanism directly. The *L_ROM_* penalty in the inverse training loop was computed against the true input wt.% and therefore has zero gradient with respect to the inverse-network parameters—reimplementation against the network’s predicted modulus is the single highest-leverage improvement available. Extension to other cotton weaves, as well as other natural fiber/polymer systems (e.g., flax/epoxy, hemp/PLA) would also be a natural next step. For more practice design applications, effect of inherent fiber misalignments in the textile fabrics [36] may also be considered as part of the model.

## 6. Conclusions

Vacuum-assisted PMMA impregnation of a plain weave cotton fabric in this work provided a new controllable pathway to tailor this base natural fiber material’s mechanical behavior from a soft, extensible sheet to a stiff, high-strength composite textile. The governing variable is not merely the PMMA impregnation quantity but the resulting polymer distribution within the fiber network. The work also presented a physics-aware artificial neural network framework that coupled this impregnation process with a shared-encoder forward and inverse predictive model, bidirectionally mapping composition, gravimetric porosity, and thermo-mechanical state to Young’s modulus, ultimate tensile strength, ultimate tensile strain, and toughness. Across the five PMMA loadings examined, we identified three distinct micromechanical behaviors: a plasticization-dominated regime at 0.1 wt.% PMMA, an inter-fiber bridging regime at 0.5–1.0 wt.% PMMA in which the modulus, strength, and toughness peak at 1.40 GPa, 67 MPa, and 1263 MPa respectively, and a polymer-dominant (matrix-rich) inclusion regime at 5 wt.% PMMA. The introduced thermo-mechanical consistency feature Δ*w* was found to be as high as 1.452 at 0.1 wt.% PMMA versus ≤1.001 at all other compositions, giving the trained network an interpretable physical signal in addition to the explicit ROM and Gordon–Taylor equations employed as part of regularization. The statistical analyses confirmed that the improvements from physics regularization are not artifacts of random variation but represent statistically significant and practically meaningful gains, with large effect sizes (Cohen’s d > 0.8) for all key comparisons. The presented bidirectional ANN framework may be used in practice, for the given composite system, as a process/material design tool through its forward/inverse inferences.

## Figures and Tables

**Table 1:** Solution preparation and gravimetric uptake during vacuum-assisted PMMA impregnation of cotton coupons; average measured values reported; note that at 0.0 wt.% PPMA, the porosity corresponds to the inherent yarn spacing in the dry fabric.

Nominal PMMA (wt.%)	Acetone (mL)	Cotton Mass Before (g)	Cotton Mass After (g)	Δm (g)	PMMA in Composite (wt.%)	Areal Density (g·m^−2^)	Thickness t (cm)	Porosity ϕ (–)
0.0	200	2.61	2.61	—	—	76.8	0.020	0.623
0.1	200	2.63	2.69	0.06	2.23	86.1	0.024	0.674
0.5	200	2.62	2.84	0.22	7.75	104.7	0.030	0.720
1.0	200	2.64	2.92	0.28	9.59	124.8	0.036	0.759
5.0	200	2.62	3.02	0.40	13.25	79.2	0.022	0.588

**Table 2:** Forward and inverse models’ performance on the specimen-level held-out evaluation set (*N* = 200 per target). Bootstrap intervals are reported at both the record level and the (specimen) group level.

Target	MAPE (%)	95% CI (Record)	95% CI (Group)	MAE	RMSE	Bias	Pearson r
PMMA wt.%	0.116	[0.047, 0.203]	[0.069, 0.241]	6.15 × 10^−4^	1.10 × 10^−3^	−2.15 × 10^−4^	0.999
*E* (MPa)	0.024	[0.020, 0.029]	[0.022, 0.034]	0.020	0.029	+0.013	0.998
*ε*_UTS_ (No Unit)	0.094	[0.072, 0.119]	[0.080, 0.131]	0.072	0.119	<10^−3^	0.997
*U*_t_ (MPa)	0.076	[0.056, 0.096]	[0.062, 0.108]	0.056	0.096	−0.008	0.989
*σ*_UTS_ (MPa)	0.093	[0.074, 0.112]	[0.079, 0.122]	0.074	0.112	−0.007	0.997

## Data Availability

The original contributions presented in this study are included in the article. Further inquiries can be directed to the corresponding author.

## Supplementary Materials

The following supporting information can be downloaded at: https://www.mdpi.com/article/10.3390/ma19112387/s1, Figure S1: Two-step PMMA dissolution and vacuum-impregnation schematic. (A) PMMA dissolution in acetone at 40 °C with mechanical stirring; (B) impregnation of the cotton substrate, air drying, vacuum impregnation, and post-drying; Figure S2: Full engineering stress–strain curves for all PMMA compositions (0, 0.1, 0.5, 1.0 and 5.0 wt.%) with five representative replicates per composition overlaid on a common strain axis; Figure S3: Porosity-exponent calibration. (A) Nonlinear least-squares fit of the porosity attenuation factor (1 − ϕ)^n^ in the modified rule-of-mixtures (Equation (2)); The fitted exponent n = 0.213 +/− 0.3 is shown with a sensitivity band spanning 95% bootstrap CI 0.04–0.48 for n. (B) Predicted versus measured elastic modulus across PMMA compositions for different n values, bounding the ROM prediction uncertainty to +/−8 % of the measured modulus; Figure S4: Training and validation learning curves for the physics-aware ANN. (A) Forward model and (B) inverse model. Loss (MAPE plus physics penalty) plotted on a logarithmic scale versus training epoch; convergence within 250 epochs in every replicate; Figure S5: (a) Per-property held-out MAPE for Phys-ON (features only) vs Phys-OFF (features + L_ROM/L_Tg penalties); Phys-ON wins on three of four targets. (b) 5-fold cross-validation mean ± std for both configurations; train and validation bars are indistinguishable, confirming the absence of overfitting. See Tables S19, S20; Figure S6: Inverse-model training curves under three independent random seeds (42, 123, 456). (a) Per-epoch train and validation MAPE; train and validation track tightly within every seed. (b) Seed-averaged curves with min–max envelopes—final validation MAPE varies between 3.04% and 3.19%, confirming robustness to initialization; Table S1.1: Nomenclature; Table S1: Solution preparation and gravimetric uptake during vacuum-assisted PMMA impregnation of cotton coupons; Table S2: Engineering parameters from Instron 5969 tensile testing for cotton/PMMA composites (mean over n ≥ 3 replicates); Table S3: Forward-model prediction accuracy and reliability intervals (specimen-level held-out evaluation set, N = 200). MAPE 95% CI (record-level): 0.047–0.203%; group-level: 0.069–0.241%; Table S4: Error-bias and distributional diagnostics for forward and inverse models on the held-out evaluation set; Table S5: Property-wise inverse prediction performance (N = 200 per property) on the specimen-level held-out evaluation set; Table S6: Unified ANN architecture and training hyperparameters; Table S7: Physics-penalty impact on the forward model (specimen-level evaluation set, N = 200); Table S8: Physics-penalty impact on inverse per-property performance with 95% bootstrap CIs; Table S9: Inverse-model per-property validation summary on the full validation set (N ≈ 1000 per property); Table S10: Composition-resolved DSC parameters from second-heat scans (10 °C/min, N_2_ atmosphere); Table S11: Hyperparameter sensitivity sweep (mean ± SD across five seeds); Table S12: Record-level vs group-level 95% bootstrap percentile intervals (2,000 resamples); Table S13: Forward-task comparison with classical baselines (extended). All models trained on the same specimen-level GroupShuffleSplit; Ridge and Random Forest use the same engineered features as the MLP for fairness; Table S14: Inverse-model residual diagnostics on the held-out evaluation set (N = 200 per property). All biases are statistically indistinguishable from zero; residuals after log transform are approximately normal; Levene and Kruskal–Wallis tests detect no heteroscedasticity or median shift across composition bins; Table S15: Per-coupon gravimetric porosity ϕ, mechanical inputs, and engineered-feature outputs used in the gravimetric calibration; Table S16: Fitted porosity exponent n and bootstrap uncertainty (B = 2000 resamples); Table S17: Inverse-model 5-fold cross-validation, per fold. Phys-ON = features only; Phys-OFF = features + L_ROM/L_Tg penalties; Table S18: Inverse-model 5-fold CV summary (mean ± std); Table S19: Forward-model 5-fold cross-validation, per fold. Phys-OFF (with L_ROM/L_Tg penalties) is unstable under the ϕ/n calibration—penalty saturates because (1 − ϕ) ^0.213^ is nearly constant across coupons; Table S20: Forward-model 5-fold CV summary. Only Phys-ON is meaningful under this calibration; Table S21: Per-property inverse-model MAPE on the 5-fold cross-validation set (post-gravimetric retrain). Values are 5-fold means; Phys-ON and Phys-OFF row-averages match Table S18; Table S22: Phys-ON vs Phys-OFF gap summary across both networks. Combines Table S18 (inverse) and Table S20 (forward) for cross-network comparison.
