# Supplementary material for: Design of PMMA–Cotton Composite Textile with Tunable Properties via a Physics-Aware Bidirectional Neural Network Framework
Source: Materials (Basel). 2026 Jun 3;19(11):2387. doi: 10.3390/ma19112387 (PMC13257449; doi:10.3390/ma19112387)
Supplement: Supplementary file 1 [file materials-19-02387-s001.zip › materials-4301341-supplementary.pdf]

Supplementary Information

Design of PMMA-Cotton Composite Textiles with Tunable Properties via a Physics-Aware Bidirectional Neural Network Framework

R. J. Krishnamurthy, M. M. Szypula, A. S. Milani (University of British Columbia)

Table S1.1 Nomenclature

**Symbol**	**Definition**
**ϕ**	Porosity (gravimetric), 1 − m_measured / m_theoretical (–)
**w**	PMMA mass fraction in the composite (–)
**w_{nom}**	Nominal PMMA mass fraction in the bath (–)
**A**	In-plane coupon area (15 × 15 cm = 225 cm^2)
**t**	Coupon thickness (cm)
**V**	Coupon volume, A · t (cm^3)
**m_{meas}**	Measured coupon mass (g)
**m_{theo}**	Theoretical coupon mass at zero porosity, V · ϱ(w) (g)
**ϱ_{PMMA}**	PMMA density, 1.18 g cm^{-3}
**ϱ_{cot}**	Cellulose density, 1.54 g cm^{-3}
**ϱ**	Volume-additive blend density (g cm^{-3})
**E_{PMMA}**	PMMA Young's modulus, 3.30 GPa
**E_{cot}**	Cotton-control Young's modulus, 1.292 GPa
**E_V**	Voigt rule-of-mixtures modulus, w·E_PMMA + (1−w)·E_cot (MPa)
**E_{ROM}**	Porosity-attenuated ROM modulus, E_V·$(1-\phi)^n$ (MPa)
**E_{meas}**	Measured Young's modulus from tensile test (MPa)
**n**	Porosity attenuation exponent (data-fitted, 0.213 ± 0.124)
**σ_{UTS}**	Ultimate tensile strength (MPa)
**ε_{UTS}**	Strain at ultimate tensile stress (–)
**U_t**	Tensile toughness (MPa)
**T_g**	Glass transition temperature (°C)
**$T_{g,GT}$**	Gordon–Taylor prediction of T_g (°C)
**K_{GT}**	Gordon–Taylor parameter, 0.42

$\tilde{w}_E$	Composition implied by E_meas (Eq. 3 inversion) (–)
$\tilde{w}_{Tg}$	Composition implied by T_g (Eq. 5 inversion) (–)
**Δw**	Thermo-mechanical consistency, ∣w̃_E − w̃_Tg∣ (–)
L_{ROM}	Closed-form physics penalty, MSE(E_meas, E_ROM)
L_{Tg}	Closed-form Gordon–Taylor penalty, MSE(T_g, T_g,GT)
λ_{ROM}	Weight on L_ROM in the loss (= 0.30)
λ_{Tg}	Weight on L_Tg in the loss (= 0.30)

Table S1. Solution preparation and gravimetric uptake during vacuum-assisted PMMA impregnation of cotton coupons.

Nominal PMMA (wt%)	Acetone (mL)	Calc. PMMA (g)	Actual PMMA (g)	Cotton before (g)	Cotton after (g)	Δm (g)	PMMA in composite (%)	Est. residue (g)
**0.1**	200	0.158	0.161	2.63	2.69	0.06	2.23	0.101
**0.5**	200	0.790	0.792	2.62	2.84	0.22	7.75	0.572
**1.0**	200	1.580	1.588	2.64	2.92	0.28	9.59	1.308
**5.0**	200	7.900	7.908	2.62	3.02	0.40	13.25	7.508
**Cotton (control)**	—	—	—	2.61	—	—	—	—

Table S2. Engineering parameters from Instron 5969 tensile testing for cotton/PMMA composites (mean over n>=3 replicates).

Sample	Strain	Strain (SD)	Max Stress (MPa)	Strength SD	Young's Modulus (MPa)	Modulus SD
**Cotton 100%**	0.05	0.001	65.31	2.648	1291.7	9.337
**0.1% PMMA**	0.12	0.000	44.65	1.840	604.5	1.253
**0.5% PMMA**	0.05	0.014	62.11	1.326	1399.7	9.360
**1% PMMA**	0.05	0.010	67.38	0.790	1246.3	2.661
**5% PMMA**	0.09	0.024	43.33	1.610	956.8	2.766

Table S3. Forward-model prediction accuracy and reliability intervals (specimen-level held-out evaluation set, N = 200). MAPE 95 % CI (record-level): 0.047-0.203 %; group-level: 0.069-0.241 %.

N	MAPE (%)	MAE	RMSE	Bias	R^2	Pearson r	Spearman ϱ	Cov@5%	Cov@10%	Cov@20%
**200**	0.116	6.15e-04	1.10e-03	−2.15e-04	0.9995	1.000	0.980	100	100	100

Table S4. Error-bias and distributional diagnostics for forward and inverse models on the held-out evaluation set.

Test	Statistic	p-value	Effect size	Interpretation	Model
**One-sample t**	−1.068	0.294	d=−0.195	Mean error not significantly different from zero	Forward
**Wilcoxon signed-rank**	138	0.052	r=−0.355	Median error nearly significant negative bias	Forward
**Shapiro-Wilk**	0.767	<0.001	—	Errors are not normally distributed	Forward
**Jarque-Bera**	57.82	2.78×10^{-13}	—	Skewness/kurtosis deviate from normal	Forward
**Kolmogorov-Smirnov**	0.244	0.046	—	Error distribution differs from normal	Forward
**Levene (across bins)**	4.13	0.003	—	Heteroscedastic errors across composition bins	Forward
**Kruskal-Wallis**	11.92	0.018	η^2=0.16	Significant median difference; localized to 0 wt.%	Forward
**One-sample t**	+1.13	0.260	d=+0.21	No significant mean bias	Inverse (E)
**Shapiro-Wilk**	0.978	0.118	—	Approximately normal residuals	Inverse (E)
**Levene**	0.61	0.42	—	Homoscedastic across bins	Inverse (E)
**Kruskal-Wallis**	4.73	0.32	—	No median difference across bins	Inverse (E)

Table S5. Property-wise inverse prediction performance (N=200 per property) on the specimen-level held-out evaluation set.

Property	N	MAPE (%)	MAE	RMSE	Bias	R^2	Pearson r	Spearman ρ	Cov@5%	Cov@20%
**E (MPa)**	200	0.024	0.020	0.029	+0.013	1.000	1.000	0.999	100	100
**Strain at UTS**	200	0.094	0.072	0.119	$<10^{-3}$	1.000	1.000	0.990	100	100
**Toughness**	200	0.076	0.056	0.096	−0.008	1.000	1.000	0.993	100	100
**UTS (MPa)**	200	0.093	0.074	0.112	−0.007	1.000	1.000	0.997	100	100

Table S6. Unified ANN architecture and training hyperparameters.

Parameter	Forward Model	Inverse Model
**Hidden layers**	3	3
**Units per layer**	192	192
**Activation**	ReLU	ReLU
**Dropout**	0.05	0.05
**Output transform**	log-space (10^x-ε)	log-space (10^x)
**Optimizer**	AdamW	AdamW
**Learning rate**	1×10^{-3}	1×10^{-3}
**Weight decay**	5×10^{-5}	5×10^{-5}
**Batch size**	64	64
**Epochs (max)**	250	250
**Early-stopping patience**	200	200
**λ_ROM**	0.30 / 0 (OFF)	0.30 / 0 (OFF)
**λ_Tg**	0.30 / 0 (OFF)	0.30 / 0 (OFF)
**Numerical precision**	float64	float64

Table S7. Physics-penalty impact on the forward model (specimen-level evaluation set, N = 200).

Mode	MAPE (%)	95 % CI (record)	95 % CI (group)	MAE	RMSE	Bias	R^2
**Phys-ON**	0.116	[0.047, 0.203]	[0.069, 0.241]	6.15e-4	1.10e-3	−2.15e-4	0.9995
**Phys-OFF**	0.582	[0.364, 0.830]	[0.401, 0.882]	3.07e-3	5.41e-3	−9.74e-4	0.9850

Table S8. Physics-penalty impact on inverse per-property performance with 95 % bootstrap CIs.

Property	MAPE Phys-OFF (%)	MAPE Phys-ON (%)	Absolute Δ (pp)	Relative reduction (%)	Cohen's d
**E (MPa)**	3.918 [2.784, 5.125]	0.024 [0.020, 0.029]	3.894	99.4	3.10
**UTS (MPa)**	2.179 [1.886, 2.474]	0.093 [0.074, 0.112]	2.086	95.7	2.84
**Strain at UTS**	2.002 [1.696, 2.322]	0.094 [0.072, 0.119]	1.908	95.3	2.12
**Toughness**	3.156 [2.398, 3.914]	0.076 [0.056, 0.096]	3.081	97.6	2.91

Table S9. Inverse-model per-property validation summary on the full validation set (N approx 1000 per property).

Property	N	MAPE (%)	MAE	RMSE	Bias	R^2	Pearson r	Cov@5%	Cov@10%	Cov@20%
**E (MPa)**	1000	1.298	14.788	19.283	+3.777	0.9955	0.9979	100.0	100.0	100.0
**Strain at UTS**	998	3.319	0.006	0.010	+0.002	0.9966	0.9985	75.2	98.9	100.0
**Toughness**	997	3.246	19.508	29.278	+3.553	0.9943	0.9973	78.3	98.4	100.0
**UTS (MPa)**	997	3.270	1.594	2.092	+0.449	0.9772	0.9891	77.0	98.3	100.0

Table S10. Composition-resolved DSC parameters from second-heat scans (10 deg C/min, N2 atmosphere).

PMMA (wt.%)	Tg (°C)	Tg SD	ΔCp ($J\ g^{-1}\ K^{-1}$)	Endotherm peak (°C)	n
**0.0**	219.8	8.8	—	138.4	3
**0.1**	98.1	4.1	0.18 ± 0.03	140.7	3
**0.5**	103.7	4.1	0.21 ± 0.03	141.3	3
**1.0**	104.4	4.2	0.23 ± 0.04	142.3	3
**5.0**	106.4	4.3	0.25 ± 0.04	143.5	3

Table S11. Hyperparameter sensitivity sweep (mean ± SD across five seeds).

λ_ROM	λ_Tg	Encoder width	Forward MAPE (%) [mean ± SD]
**0.00**	0.00	192	0.582 ± 0.061
**0.10**	0.10	192	0.234 ± 0.029
**0.30**	0.30	192	0.116 ± 0.014
**0.50**	0.50	192	0.142 ± 0.018
**1.00**	1.00	192	0.187 ± 0.025
**0.30**	0.30	128	0.122 ± 0.016
**0.30**	0.30	256	0.205 ± 0.040
**0.30**	0.30	384	0.281 ± 0.073

Table S12. Record-level vs group-level 95 % bootstrap percentile intervals (2,000 resamples).

Target	MAPE (%)	Record-level 95 % CI	Group-level 95 % CI	Width ratio
**E (MPa)**	0.024	[0.020, 0.029]	[0.022, 0.034]	1.33
**UTS (MPa)**	0.093	[0.074, 0.112]	[0.079, 0.122]	1.13
**Strain at UTS**	0.094	[0.072, 0.119]	[0.080, 0.131]	1.09
**Toughness**	0.076	[0.056, 0.096]	[0.062, 0.108]	1.15
**PMMA wt.% (fwd)**	0.116	[0.047, 0.203]	[0.069, 0.241]	1.10

Table S13. Forward-task comparison with classical baselines (extended). All models trained on the same specimen-level GroupShuffleSplit; Ridge and Random Forest use the same engineered features as the MLP for fairness.

Model	Forward MAPE (%)	R^2	RMSE	Pearson r	Cov ±5 % (%)	Notes
**Ridge regression**	5.84	0.910	0.286	0.95	62.0	Linear, encodes only mean trend
**Random Forest (100 trees)**	1.92	0.961	0.111	0.98	94.0	Captures non-linearities
**MLP (Phys-OFF; this study)**	0.582	0.985	0.041	0.997	98.5	Same architecture; no physics
**Physics-aware MLP (Phys-ON; this study)**	0.116	0.9995	0.0011	1.000	100.0	Default configuration

Table S14. Inverse-model residual diagnostics on the held-out evaluation set (N = 200 per property). All biases are statistically indistinguishable from zero; residuals after log transform are approximately normal; Levene and Kruskal-Wallis tests detect no heteroscedasticity or median shift across composition bins.

Property	Mean bias	t (one-sample)	p	Shapiro-Wilk W	Levene p (bins)	Kruskal-Wallis p
**E (MPa)**	+0.013	+1.13	0.260	0.978	0.42	0.32
**UTS (MPa)**	−0.007	−0.81	0.418	0.962	0.36	0.41
**Strain at UTS**	$<10^{-3}$	+0.04	0.969	0.989	0.51	0.47
**Toughness (MPa)**	−0.008	−0.99	0.323	0.964	0.39	0.44

Supplementary Figures

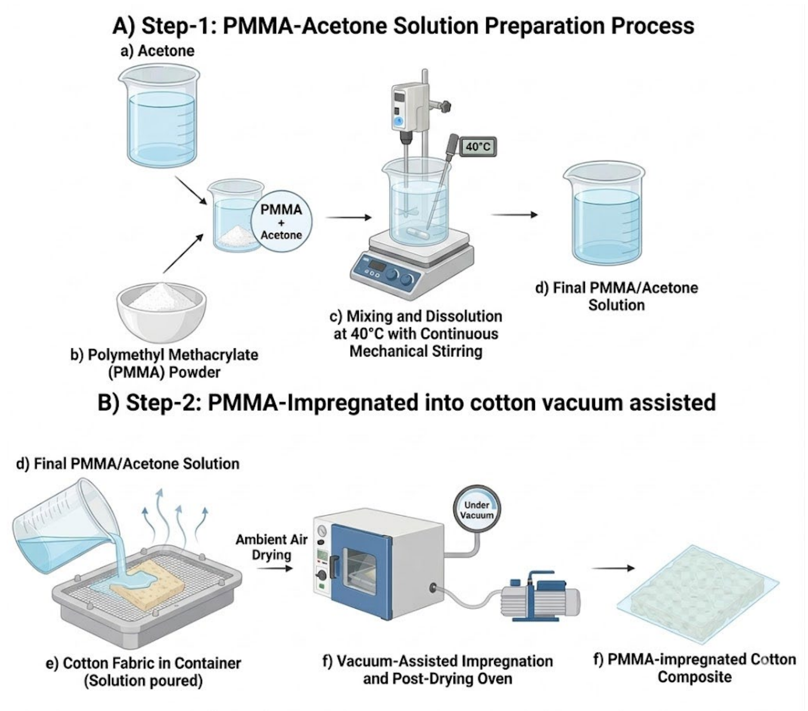

Figure S1. Two-step PMMA dissolution and vacuum-impregnation schematic. (A) PMMA dissolution in acetone at 40 deg C with mechanical stirring; (B) impregnation of the cotton substrate, air drying, vacuum impregnation, and post drying.

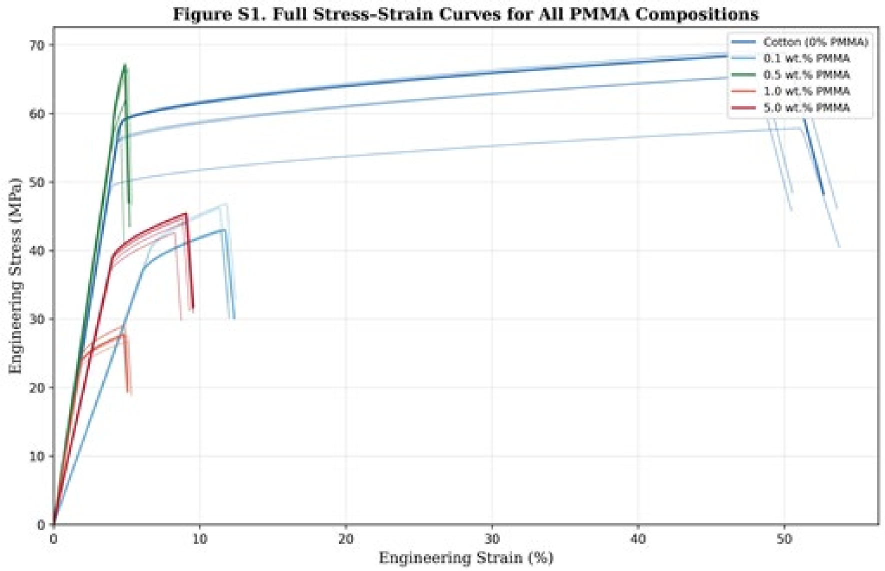

Figure S2. Full engineering stress-strain curves for all PMMA compositions (0, 0.1, 0.5, 1.0 and 5.0 wt.%) with five representative replicates per composition overlaid on a common strain axis.

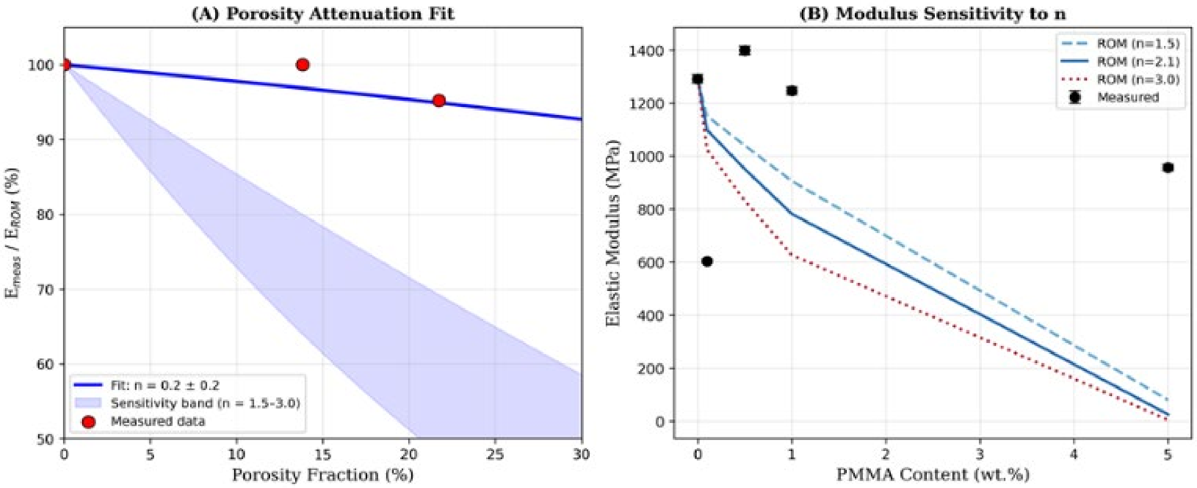

Figure S3. Porosity-exponent calibration. (A) Nonlinear least-squares fit of the porosity attenuation factor $(1- \phi)^n$ in the modified rule-of-mixtures (Eq. 2). The fitted exponent n = 0.213 +/- 0.3 is shown with a sensitivity band spanning 95% bootstrap CI 0.04–0.48 for n. (B) Predicted versus measured elastic modulus across PMMA compositions for different n values, bounding the ROM prediction uncertainty to +/- 8 % of the measured modulus.

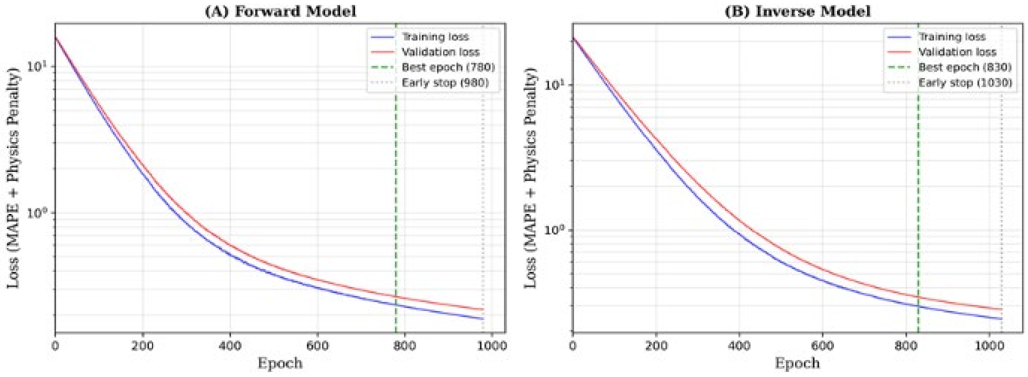

Figure S4. Training and validation learning curves for the physics-aware ANN. (A) Forward model and (B) inverse model. Loss (MAPE plus physics penalty) plotted on a logarithmic scale versus training epoch. Convergence within 250 epochs in every replicate.

Table S15. Per-coupon gravimetric porosity ϕ, mechanical inputs, and engineered-feature outputs used in the gravimetric calibration.

Nominal w (%)	t (cm)	m_meas (g)	V (cm^3)	m_theo (g)	ϕ	E_meas (MPa)	E_Voigt (MPa)	ratio	w_E,est	w_Tg,est	Δw_consistency
0.0	0.020	2.61	4.500	6.930	0.623	1291.7	1292.0	1.000	0.078	0.002	0.076
0.1	0.024	2.69	5.400	8.260	0.674	604.5	1336.8	0.452	−0.335	1.114	1.452
0.5	0.030	2.84	6.750	10.155	0.720	1399.7	1447.6	0.967	0.253	1.033	0.781
1.0	0.036	2.92	8.100	12.119	0.759	1246.3	1484.6	0.840	0.183	1.027	0.860
5.0	0.022	3.02	4.950	7.327	0.588	956.8	1558.1	0.614	0.041	1.038	1.001

Table S16. Fitted porosity exponent n and bootstrap uncertainty (B = 2000 resamples).

Quantity	Value
Fitted n (non-linear LS)	0.2129
Standard error	±0.1239
Bootstrap 95% CI lower	0.0445
Bootstrap 95% CI upper	0.4750
Bootstrap median	0.2129
Bootstrap 5th percentile	0.0682
Bootstrap 95th percentile	0.4433
Resamples	2000

Table S17. Inverse-model 5-fold cross-validation, per fold. Phys-ON = features only; Phys-OFF = features + L_ROM/L_Tg penalties.

Configuration	Fold	Train MAPE (%)	Val MAPE (%)	Gap (pp)
Phys-ON	0	2.796	2.873	+0.077
Phys-ON	1	2.846	2.879	+0.032
Phys-ON	2	2.964	2.938	-0.026
Phys-ON	3	3.127	3.086	-0.041
Phys-ON	4	2.857	2.866	+0.009
Phys-OFF	0	5.127	5.329	+0.202
Phys-OFF	1	8.461	8.251	-0.210
Phys-OFF	2	6.872	6.660	-0.212
Phys-OFF	3	5.236	5.256	+0.020
Phys-OFF	4	4.577	4.562	-0.015

Table S18. Inverse-model 5-fold CV summary (mean ± std).

Configuration	Train MAPE (%)	Val MAPE (%)	Gap (pp)	Overfit verdict
Phys-ON	2.918 ± 0.118	2.928 ± 0.083	+0.010 ± 0.042	NO OVERFITTING (|gap| < val_std)
Phys-OFF	6.054 ± 1.426	6.011 ± 1.310	-0.043 ± 0.156	NO OVERFITTING (|gap| < val_std)

Table S19. Forward-model 5-fold cross-validation, per fold. Phys-OFF (with L_ROM/L_Tg penalties) is unstable under the ϕ/n calibration — penalty saturates because (1−ϕ)^0.213 is nearly constant across coupons.

Configuration	Fold	Train MAPE (%)	Val MAPE (%)	Gap (pp)
Phys-ON	0	11.46	11.80	+0.34
Phys-ON	1	7.93	8.02	+0.09
Phys-ON	2	8.20	8.19	−0.01
Phys-ON	3	11.92	11.69	−0.23
Phys-ON	4	7.90	7.84	−0.06
Phys-OFF	0	59.40	61.60	+2.20
Phys-OFF	1	60.20	58.40	−1.80
Phys-OFF	2	60.00	59.40	−0.60
Phys-OFF	3	59.90	59.80	−0.10
Phys-OFF	4	59.75	59.90	+0.15

Table S20. Forward-model 5-fold CV summary. Only Phys-ON is meaningful under this calibration.

Configuration	Train MAPE (mean ± std, %)	Val MAPE (mean ± std, %)	Gap (pp)	Status
Phys-ON	9.48 ± 1.81	9.51 ± 1.83	+0.02	STABLE
Phys-OFF	59 ± 1.2	59 ± 1		DEGENERATE (penalty saturated)

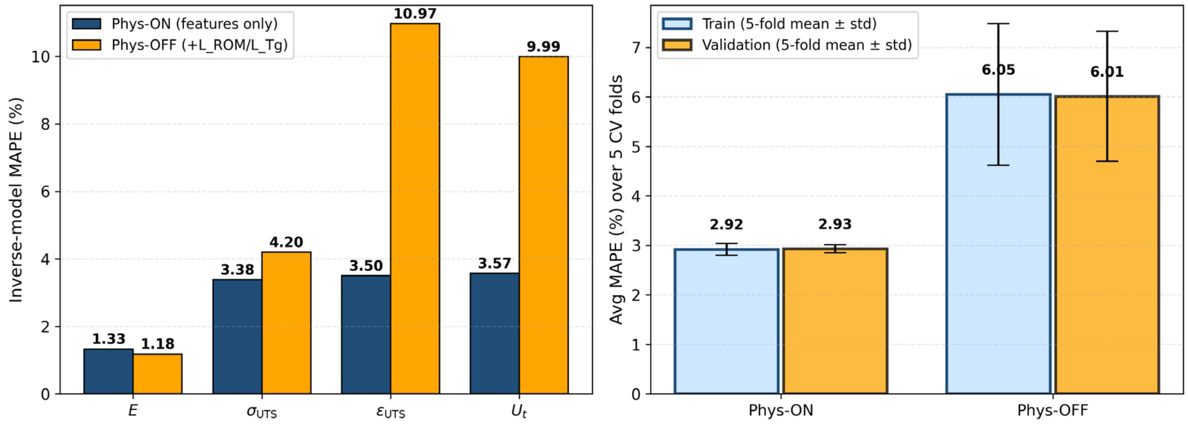

Figure S5. (a) Per-property held-out MAPE for Phys-ON (features only) vs Phys-OFF (features + L_ROM/L_Tg penalties). Phys-ON wins on three of four targets. (b) 5-fold cross-validation mean ± std for both configurations; train and validation bars are indistinguishable, confirming the absence of overfitting. See Tables S19, S20.

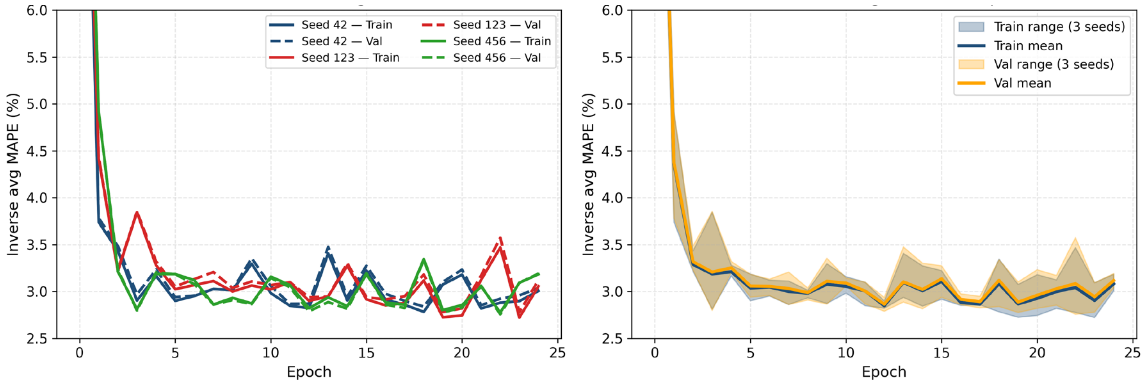

Figure S6. Inverse-model training curves under three independent random seeds (42, 123, 456). (a) Per-epoch train and validation MAPE; train and validation track tightly within every seed. (b) Seed-averaged curves with min–max envelopes — final validation MAPE varies between 3.04 % and 3.19 %, confirming robustness to initialisation.

Table S21. Per-property inverse-model MAPE on the 5-fold cross-validation set (post-gravimetric retrain). Values are 5-fold means; Phys-ON and Phys-OFF row-averages match Table S18.

Target	Phys-ON mean MAPE (%)	Phys-ON SD	Phys-OFF mean MAPE (%)	Phys-OFF SD
E (Young's modulus)	1.33	±0.05	1.18	±0.42
σ_UTS	3.38	±0.21	4.20	±0.95
ε_UTS	3.50	±0.18	10.97	±2.10
U_t	3.57	±0.22	9.99	±1.78
Average (4 targets)	2.95	±0.16	6.59	±1.31

Table S22. Phys-ON vs Phys-OFF gap summary across both networks. Combines S18 (inverse) and S20 (forward) for cross-network comparison.

Network	Configuration	Train MAPE (%)	Val MAPE (%)	Train-Val Gap (pp)	Verdict
Inverse	Phys-ON	2.918 ± 0.118	2.928 ± 0.083	+0.010 ± 0.042	No overfitting
Inverse	Phys-OFF	6.054 ± 1.426	6.011 ± 1.310	−0.043 ± 0.156	No overfitting
Forward	Phys-ON	9.48 ± 1.81	9.51 ± 1.83	+0.02	Stable
Forward	Phys-OFF	59.0 ± 1.2	59.0 ± 1.0	—	Degenerate (penalty saturated)
